# A qualitative study of the cognitive behavioral intention of patients with diabetes in rural China who have experienced delayed diagnosis and treatment

**DOI:** 10.1186/s12889-020-08636-2

**Published:** 2020-04-10

**Authors:** Hong-hong Jia, Li Liu, Gui-xia Huo, Rui-qi Wang, Yu-qiu Zhou, Li-yan Yang

**Affiliations:** 1 Department of Nursing, Harbin Medical University (Daqing), Xinyang Road No. 39, Daqing, 163319 China; 2 Endocrinology, Caofeidian Area Hospital in Hebei Province, Tangshan, 063500 China; 3 School of Nursing, Suzhou Vocational Health College, Suzhou, 215000 China

**Keywords:** Patient and treatment delay, Cognitive behavioral intention, Rural diabetes, Qualitative research

## Abstract

**Background:**

Great changes have taken place in terms of people’s lifestyles and behavioral habits. Diabetes has become a threat to human health and is the most important noncommunicable disease. More than 60% of rural diabetic patients experience delayed diagnosis and treatment. In this study, we explore the inner experience of the delayed diagnosis and treatment of patients with diabetes in rural areas and provide a reference for targeted intervention.

**Methods:**

A qualitative research design was used to examine the cognitive behavioral intention of patients in rural areas with delayed diagnosis and treatment of diabetes. Thirteen diabetes patients with delayed diagnosis and treatment were sampled with maximum variation in rural Daqing City and Tangshan City in China. The data analysis involved several levels of analysis consistent with qualitative research.

**Results:**

The following themes were relevant to diabetes patients in rural areas with delayed diagnosis and treatment delay: “Lacked knowledge of diabetes”, “Negative coping style”, “Dissatisfaction with the existing medical service” and “Influence of social support”.

**Conclusions:**

The respondents’ delayed diagnosis and treatment represent a common phenomenon. Medical personnel should provide interventions for patients and encourage them to go to the hospital on time.

## Background

Diabetes mellitus, which has become a threat to human health, is the most important noncommunicable disease [1]. In 2013, 11.6% (approximately 1140,000) of Chinese people aged above 18 reported having diabetes mellitus [2]. More than 60% of rural diabetic patients experience delayed diagnosis and treatment [3]. Without supervision, blood sugar is controlled badly, and errors in using insulin can influence disease control [4]. Only 18.8, 16.2 and 8.0% of the people aged 35 to 74 with diabetes in China’s rural areas engaged in proper awareness, treatment and control of diabetes, respectively [5]. The reasons for such low percentages include poor medical condition, low degree of education and low level of cognitive behavioral intention of residents in rural areas [6].

Delayed diagnosis and treatment of patients with diabetes have serious impacts on the quality of life and disease prognosis. Gulam showed that mild diabetes that is not treated in time may incur complications, such as infection, sepsis and gangrene, and can increase the risk of amputation and death [7]. Zubair believed that the delayed time of diagnosis and the amputation rate were significantly positively related, as delayed treatment time increases the likelihood of amputation for patients with diabetes [8]. Stone P A believed that persistent inflammation and blood glucose fluctuations may induce cardiovascular events and increase cardiovascular disease mortality, such as acute myocardial infarction and cardiomyopathy [9].

Delayed diagnosis and treatment are characterized by a delay in diagnosis and a delay in treatment. A delay in diagnosis, which is called a patient delay, occurs if the time between when a patient first observes suspicious symptoms and when the patient first visits a doctor in a medical institution spans more than 3 months [10, 11]. A delay in treatment refers to a situation in which a patient with diabetes begin to receive regular treatment more than 2 weeks after receiving the diagnosis or a situation in which a patient who is in the process of receiving treatment does not receive treatment in a timely matter [12].

Most studies of diabetes have focused on complications and influencing factors, while few have considered delayed diagnosis and treatment. China has started to attach great importance to the prevention of diabetes, but there is a lack of prehospital targeted screening and early intervention measures, with impacts on prevention, diagnosis and treatment. This study was conducted to explore the inner experience of clinical treatment and the behavioral intention concerning delayed diagnosis and treatment of patients with diabetes and provides a reference for comprehensive intervention in rural China.

## Methods

### Design

In this phenomenological qualitative research, interviews and observations were used to collect data for conventional content analysis through a qualitative descriptive method conducted by the first author. Content analysis can explain and classify textual data by considering individual cultural and contextual effects on phenomena. The final products of data analysis for content analysis are classifications and themes [13].

### Participants

This research adopts the convenience sampling method, and thirteen diabetes patients with delayed diagnosis and treatment were sampled with maximum variation in rural Daqing City and Tangshan City in China. All diabetes patients with delayed diagnosis and treatment had to meet the inclusion criteria provided by the first author to participate in the study. The inclusion criteria were having diabetes, living in a rural area and having experienced delayed diagnosis and treatment. Further information gathered from the patients interviewed included demographic background, age, gender, course of the disease, delay time of diagnosis and treatment, level of education and family income per month.

All of the included patients with delayed diagnosis and treatment were asked and agreed to participate in this research, and all were farmers.

### Data collection

The data collection was carried out from April to October 2018. In the process of the in-depth interviews, all diabetes patients with delayed diagnosis and treatment were informed of the aim of the study and agreed to participate. The respondents were encouraged to express their inner thoughts concerning the behavioral intention behind their delayed diagnosis and treatment of diabetes. All interviews were conducted in a quiet and well-lit room, comprised a face-to-face conversation between the interviewee and the interviewer, and lasted from 45 to 60 min. The researcher listened carefully and recorded the audio contents of the interview with a recording pen at the same time, and the interviewees’ behaviors and expressions were also recorded in a timely manner. After the interview, we returned the transcripts to the respondents, and they were asked to check whether the transcripts were consistent with their intention in order to improve the credibility of the results.

When information was repeated in the process of the interviews, the sample size was considered to have reached saturation. All diabetes patients with delayed diagnosis and treatment and living in rural areas spoke Chinese, and the researcher translated the interviews into English to obtain the results. The outline of the interview was as follows (Table 1).

**Table 1:** Outline of the interview

1. Please tell me what you know about diabetes.	
2. Please tell me how you were diagnosed with diabetes.	
3. Please tell me how long you waited to go to the hospital when you had symptoms of diabetes.	
4. Why did you go to the hospital for treatment the first time?	
5. What reason impacted your going to the hospital?	
6. How did you feel after being diagnosed with diabetes?	

### Data analysis

There were 2 researchers,who had nursing master’s degree, involved in coding the data. The seven-step Colaizzi phenomenology was used to analyze the interview data [14]. The qualitative research and analysis software QSR Nvivo 12.0 was used to collate and analyze the data. The contents of interviews were transcribed within 24 h after the interview, and the transcriptions were returned to the respondents to verify the text information and to improve the credibility of the results. After the interviewee verified the contents, two researchers, who continuously and repeatedly read, analyzed and coded the same textual information, compared the results with the original information and formed the final theme.

### Ethical considerations

Consent for the study was obtained from the ethics committee of Daqing campus of Harbin Medical University. Before each interview, the researcher explained the purpose and content of the study to the respondents and promised confidentiality. The participants then agreed to participate in the research and signed the informed consent. Participants had the right to refuse to participate in the research at any time without reprisal.

## Results

There were thirteen participants in this study: five were male, and eight were female. All patients had type 2 diabetes. The age of the participants was between 38 and 82 years old, and the mean age was 57.53 years. The disease course was between 1 and 20 years, the mean course of the disease was 9.1 years, the delay of diagnosis and treatment was between 0.5 and 13 years, and the mean delay of diagnosis and treatment was 6.1 years. Twelve patients had diabetes complications, 4 had diabetic foot, 7 had diabetic macular edema, 9 had diabetes complications with cardiovascular symptoms, and 2 had asymptomatic symptoms. Three of the participants were illiterate, 6 had primary school education, 2 had junior high school education and 2 had senior high school education. All of the participants were married, and four had experienced the death of a spouse. Six people had a monthly family income less than 500 RMB, 5 people had a monthly family income of 500 ~ 999 RMB, 1 person had a monthly family income of 1000 ~ 1999 RMB, and 1 person had a monthly family income of more than 2000 RMB (Table 2).

**Table 2:** Characteristics of participants

Characteristic	Number
Gender	
male	5
female	8
Age	
30–39	2
40–49	3
50–59	2
60–69	2
≥ 70	4
Educational level	
illiterate	3
primary school	6
junior high school	2
senior high school	2
Marital status	
married	9
death of a spouse	4
Confirmed time (years)	
< 3	2
3–5	3
6–10	3
> 10	5
Delay of diagnosis and treatment (years)	
Delay of diagnosis (years)	
< 1	0
1–5	2
6–10	3
> 10	1
Delay of treatment (years)	
< 1	1
1–5	3
6–10	2
> 10	1
Monthly family income	
< 500 RMB	6
500–999 RMB	5
1000–1999 RMB	1
≥ 2000 RMB	1

Data analysis led to the development of four themes and ten subthemes. The themes were “Lacked knowledge of diabetes”, “Negative coping style”, “Dissatisfaction with the existing medical service” and “Influence of social support” (Table 3).

**Table 3:** Themes and subthemes

Theme	Subthemes
Lacked knowledge of diabetes	Insufficient understanding of the disease
	Lacked awareness of early treatment
	Lack of self-control and monitoring
Negative coping style	Poor medical behavior
	Fear/Anxiety
	Uncertainty about the future
Dissatisfaction with the existing medical service	Discomfort in the medical environment and the cumbersome medical process
	Unsatisfied with the professional guidance concerning the disease
Influence of social support	Support from family and others
	Medical economic burden

### Lacked knowledge of diabetes

This theme includes three subthemes: an insufficient understanding of the disease, a lack of awareness of early treatment and lack of self-control and monitoring. Only 1 participant could accurately answer questions concerning relevant knowledge, and the remaining participants were unable to answer questions accurately.

#### Insufficient understanding of disease

Most of the participants said that they did not have basic knowledge about diabetes, symptoms associated with the disease and the seriousness of delaying the diagnosis and treatment of complications.

One participant (N11) said, “I don’t know what are symptoms of diabetes and what’s the normal blood sugar. Maybe the normal blood sugar is about 6 mmol/L.”

Another participant (N05) stated, “I am a farmer; because information is underdeveloped in the countryside, I don’t know what complications diabetes has.”

The third participant (N01) said, “I didn’t know about diabetes before I had this disease, I paid attention to this information after I was sick. If I had known I was a diabetes patient, I would have gone to the hospital early.”

#### Lacked awareness of early treatment

Judgment of the severity of disease can have an effect on patients’ health behavior decisions. Two respondents thought that going to the hospital sooner or later would have no effect on the treatment of the disease. Four participants thought that if the disease did not affect normal life, it did not need treatment.

One participant (N08) said, “Diabetes is a chronic disease, it doesn’t matter that you go to hospital sooner or later.”

Another participant (N12) stated, “Everyone says that diabetes is a cancer that you can’t die from. If you are comfortable, then you don’t need to see a doctor.”

A third interviewee (N10) said, “I didn’t need to be hospitalized after the diagnosis of diabetes, I just went home and controlled it by myself. And I always thought that diabetes was ok. I was diagnosed with diabetes five years ago, and I was hospitalized for the first time because I fainted.”

#### Lack of self-control and monitoring

The patients’ effective self-health management involved a long and difficult process. Some people did not accept regular treatment, and they used folk prescriptions and delayed treatment.

One participant (N02) said, “I took the medicine, I still felt that I was bad, so I didn’t continue to take the medicine.”

Another participant (N07) stated, “Usually, I have time to try to walk for exercise, because it is difficult to control my diet. I feel no one can control their diet so strictly.”

A third participant (N09) said, “At first, the doctor told me that I should take medicine and have a strict diet, but I could not do it. I took folk prescription drugs for nearly three months, but it was no use, and I felt it was more and more serious, then I went to the hospital.”

### Negative coping style

This theme includes three subthemes: poor medical behavior, fear/anxiety and uncertainty about the future. All participants were not positive about going to the hospital for various reasons.

#### Poor medical behavior

The interviewees had a weak awareness of medical treatment in general. Two respondents said that they did not go to the hospital if diabetes mellitus had no effect on their normal life.

One participant (N08) said, “We live in the countryside and are busy every day, I don’t pay attention to myself, and I never go to the hospital.”

Another participant (N10) stated, “I had symptoms of diabetes early, but I didn’t want to come to the hospital; when I had an examination, my blood sugar was 23.5 mmol/L.”

Two respondents said that they were in good health and did not engage in treatment by themselves. Most people did not have good habits, and they thought it was trouble and a waste of time to go to the hospital.

One participant (N06) said, “I still feel very young, and the chronic disease is not going to heal, so I never want to go to the hospital; I can control the disease at home.”

#### Fear/anxiety

After some patients were diagnosed with diabetes, they were fearful and refused to discuss their diabetes with others.

One participant (N11) said, “Everybody said diabetes was difficult to cure, but people couldn’t die from diabetes. So I felt fear in the face of the disease, and I didn’t want to let others know that I had diabetes and I didn’t go to the hospital.”

Another participant (N04) stated, “I don’t want to be in the hospital because it’s too much trouble, I’m afraid of family trouble, and I also fear missing work.”

The patients with diabetes felt too much great worry and anxiety about the care of their children and families.

A married woman (N04) said, “I was worried about my child, I was afraid nobody would take care of him while I was in the hospital, and it would have an effect on his studies.”

Two respondents said that the hospital environment would produce anxiety and irritability. One participant (N13) said, “When I came to the hospital, I felt depressed, bad tempered and agitated.”

#### Uncertainty about the future

Most of the patients had no confidence in the treatment of diabetes. Their uncertainty about the future led to delayed treatment.

A patient (N04) with badly controlled blood sugar said, “I don’t know whether I can control my blood sugar or not, sometimes I had no confidence.”

Another participant (N09) stated, “My current total blood sugar is above 10, now my legs are itchy. I don’t know whether going to the hospital and spending money can cure me or not.”

### Dissatisfaction with existing medical services

All participants believed that the hospital environment was of poor quality. This is the main reason most people with diabetes were reluctant to seek help.

#### Discomfort in the medical environment and the cumbersome medical process

The medical environment is not comfortable, and the poor medical services provided can directly affect the patient’s medical behavior. Three respondents said they were not satisfied with the medical environment of the hospital.

One participant (N02) said, “The environment of the hospital was poor and not comfortable. There were too many patients, and they did not have good rest in the hospital.”

Another participant (N03) stated, “I think the hospital conditions do not meet the standard in my mind, there were not enough beds in the hospital, and extra beds were in the corridors. In particular, I didn’t want to the hospital.”

A third participant (N08) said, “I think that the process of seeing a doctor was too much. The fee was paid on another floor, seeing a doctor meant running around all morning, a person can’t see the doctor.”

#### Professional guidance provided about the disease

Three respondents hoped to obtain professional knowledge to guide the treatment of their disease.

One participant (N03) said, “We don’t know what diabetes is, I didn’t even know when I was sick. When I don’t feel strong enough, then I’ll go to the community hospital, but that’s no medical condition.”

Another participant (N05) stated, “Information in rural areas is developed, but I don’t know where to go to understand diabetes. Even if I’m sick, I don’t know when to see a doctor.”

### Influence of social support

Social support is very important for people with diabetes, and patients with diabetes who have good social support have higher levels of cognitive behavioral intention.

#### Support from family and others

The majority of patients with diabetes learn about the disease from family, friends and people around them. Family members’ lack of medical knowledge will lead to delayed treatment. Some respondents went to the hospital at the urging of those around them.

A patient (N03) whose blood sugar was 19.8 mmol/L said, “I don’t want to be in the hospital, but my daughter always advised me to go to the hospital.”

Another participant (N05) stated, “I didn’t take medicine after illness, but I heard she had to take medicine to control her blood sugar, so I just came to the hospital.”

A patient (N06) whose diagnosis was delayed by one year said, “People say that diabetes is a chronic disease. I didn’t need to go to the hospital for treatment for a long time. My diet and exercise could control the blood sugar, I received these effects, and so I didn’t go to the hospital.”

#### Medical economic burden

Three of the respondents expressed that they did not want to go to the hospital because of the heavy economic burden.

One participant (N05) said, “I had to spend money when I went to the hospital. I spent thousands on an optical inspection. I can’t afford it.”

Another participant (N09) stated, “Because of the high cost of the treatment of diabetes, although the national subsidy exempts some treatment. But I still can’t solve the problem of diabetes drug costs.”

## Discussion

This is the first qualitative study specifically aimed at exploring the inner experience of the clinical treatment of diabetic patients with delayed diagnosis and treatment.

### Improving the understanding of diabetes and enhance awareness and education

Relative to urban areas, diabetes health services for the rural patients in China are poor, which could be one of the reasons for delayed diagnosis and treatment in rural areas. Because rural patients tend to have a low education level, diabetes health education resources are scarce, resulting in a lack of knowledge of diabetes.

The respondents said that before they were ill, they did not take the initiative to understand diabetes, and they did not know the first symptoms of diabetes. Eventually, this led to delays in diagnosis and treatment. The respondents did not understand the importance of early treatment for diabetes and ignored self-health management. Poor blood sugar control consciousness leads to disease development and complications involving other organs. Many people’s understanding of diabetes is flawed, which results in treatment resistance. This may be due to atypical early symptoms of diabetes, which most people cannot recognize. Second, people may lack knowledge of diabetes treatment. Knowledge about diabetes has the strongest effect on health behavior, and the higher the degree of knowledge is, the better prevention and control behaviors an individual engages in [15]. Therefore, we should provide education about health, awareness and effective control of blood sugar and improved treatment for patients who have been diagnosed with diabetes [16]. People with a family history of diabetes, centripetal obesity, dyslipidemia, and lack of physical activity represent a key intervention object [17]. Medical institutions can provide free screening for diabetes, encourage people to actively seek medical attention and improve diabetes prevention awareness.

### Establish positive coping styles and motivate treatment

This study found that most of the respondents did not have good habits, and they responded negatively in the face of early symptoms. They thought their condition would improve through taking their own medication or resting more. The result was that when their symptoms affected normal life, the patients were forced to seek medical treatment. Some of the respondents did not understand the state of their body and considered themselves healthy, even delaying treatment for 16 years, and when they went to the hospital for medical treatment, they had serious complications. To address these habitual negative responses, we should first change patients’ negative medical attitudes. Zhang Cang found that a negative attitude towards medical treatment is the main reason for delayed treatment [18]. Therefore, nurses should inspire patients to actively engage in treatment, strengthen the importance of early treatment and improve patients’ self-efficacy.

### Effective emotional and social support

People with diabetes experience or whose friends work in medical-related fields engage in better medical treatment behavior and have less fear and anxiety concerning the disease. Therefore, good social support can eliminate adverse psychological reactions and promote positive treatment. Studies have shown that a good role model or a good medical treatment atmosphere can promote patient health decisions; the absence of these factors leads to delayed diagnosis and treatment [19]. Therefore, nurses should strengthen the related health guidance given to patients, provide disease knowledge education, reduce people’s fear of seeing a doctor and facing the disease, and encourage patients to see a doctor [20]. At the same time, the medical staff should establish a good trust relationship with the patient, give patients great respect, and encourage patients to adopt a positive attitude towards treatment and have more confidence in continuing treatment. After patients leave medical institutions, medical staff should keep in touch and follow up by telephone on a regular basis. Such professional guidance and supervision will more effectively promote the patients’ physical and mental health.

### Optimization of community health service and reduced economic burden

In the interviews, when mentioning reasons for their reluctance to go to the hospital, nearly half of the respondents thought that it was troublesome to go to the hospital. We conclude that treatment involves a complicated process, there are too many patients per hospital, and the patients live far away from the hospital. The best solution to this problem is to improve community health care. At present, patients’ medical understanding and patterns are changing in China, and people attach more importance to the convenience and effectiveness of treatment. The results of the study also showed that the convenience of treatment had a significant influence on residents’ medical behavior [21]. Therefore, we should enrich community medical resources, improve the technical level of medical staff, and eliminate people’s distrust for community health agencies and other problems.

In addition, this study found that people with diabetes face a heavy economic burden, which is one of the reasons for the delay of diagnosis and treatment. Research statistics reveal that hospitalized patients with diabetes (1396 cases) spend approximately 13 days in the hospital, incurring a total hospitalization expense of 6578.88 yuan, while expenses for medicine account for the greatest cost, followed by inspection, examination and treatment. The economic burden to patients with diabetes cannot be ignored. We should continue to focus on the problem, seek to perfect medical insurance for patients with diabetes and let more people enjoy treatment.

### Limitations

Because the research object of this study covers only two provinces in rural China, it is subject to bias due to region selection and research conditions. Additionally, because cultural differences exist between native English and Chinese speakers, the translation of the interviews from Chinese to English is another limitation of the study. Further strengthening of the theme extraction is necessary. Furthermore, this study was conducted on a limited number of patients with diabetes in rural China; therefore, the results cannot necessarily be generalized to all patients with diabetes in China. Because regional differences in China are relatively large, the psychological experiences of patients with delayed diagnosis and treatment of diabetes are different.

### Suggestions for future studies

Future studies may add quantitative research and engage in more in-depth investigations of the causes of the delayed diagnosis and treatment of diabetes.

## Conclusions

The findings of the current study show that 13 patients with diabetes suffered from problems of diagnosis and treatment delayed by a minimum of 2 to 13 years, and more than 90% of the respondents had complications.

People with diabetes who live in rural areas experience a serious phenomenon of delayed diagnosis, which is particularly due to poor knowledge and habits. Therefore, nurses should start with the patient’s needs and wishes, raise awareness of diabetes treatment, reduce the occurrence of delayed treatment, and improve the prevention of diabetes.
